# Esophageal schwannoma mimicking non-functional parathyroid adenoma on ^99m^Tc-sestamibi imaging: a case report

**DOI:** 10.3389/fendo.2024.1258233

**Published:** 2024-05-22

**Authors:** Roberto Fiore, Edwige Gombert, Stefano La Rosa, Vincent Dunet, Gerasimos P. Sykiotis, François Gorostidi

**Affiliations:** ^1^ Service of Endocrinology, Diabetology and Metabolism, Lausanne University Hospital and University of Lausanne, Lausanne, Switzerland; ^2^ Service of Otolaryngology and Head and Neck Surgery, Lausanne University Hospital and University of Lausanne, Lausanne, Switzerland; ^3^ Institute of Pathology, Department of Laboratory Medicine and Pathology, University of Lausanne, Lausanne, Switzerland; ^4^ Service of Pathology, Department of Medicine and Technological Innovation, University of Insubria, Varese, Italy; ^5^ Service of Diagnostic and Interventional Radiology, Lausanne University Hospital and University of Lausanne, Lausanne, Switzerland

**Keywords:** schwannoma, parathyroid, sestamibi, single-photon emission computed tomography/computed tomography, case report

## Abstract

Technetium-99m sestamibi single-photon emission computed tomography/computed tomography (^99m^Tc-sestamibi SPECT/CT) is a mainstay of the pre-operative localization of parathyroid lesions. We report here the case of a 30 year-old woman with a fortuitously discovered 2 cm cervical mass for which a parathyroid origin was originally suspected due to its retro-thyroidal localization and a personal history of nephrolithiasis. Normal serum calcium and parathyroid hormone (PTH) levels excluded primary hyperparathyroidism, raising suspicion of a non-functional parathyroid adenoma, and SPECT/CT imaging showed that the mass was ^99m^Tc-sestamibi-avid. Fine-needle aspiration (FNA) was performed; cytology was non-diagnostic but the needle washout was negative for thyroglobulin, calcitonin and PTH, arguing against a thyroidal or parathyroidal origin of the mass. Core needle biopsy revealed a schwannoma, ostensibly originating from the recurrent laryngeal nerve; upon surgical resection, it was finally found to arise from the esophageal submucosa. This case illustrates the fact that endocrinologists, radiologists, nuclear medicine, head and neck, and other specialists investigating patients with cervical masses should be aware that schwannomas need to be considered in the differential diagnosis of focal ^99m^Tc-sestamibi uptake in the neck region.

## Introduction

Technetium-99m sestamibi single-photon emission computed tomography/computed tomography (^99m^Tc-sestamibi SPECT/CT) has been reported to have a positive predictive value of >95% for the preoperative localization of parathyroid lesions in patients with primary hyperparathyroidism (1). Nevertheless, occasional false-positive results in the neck region can be due to benign or malignant thyroid disease, while various CNS tumors in the head area can show ^99m^Tc-sestamibi uptake, as is the case also for different types of benign and malignant tumors in the chest and abdomen (2). Specifically regarding schwannomas, ^99m^Tc-sestamibi has shown potential as a radionuclide for the localization of cerebellar pontine tumors (mainly acoustic schwannomas) (3). On the other hand, to the best of our knowledge, uptake of ^99m^Tc-sestamibi by a schwannoma in the neck region has never been described.

## Case presentation

### Patient information

A 30-year-old woman was referred to our endocrine outpatient clinic in January 2019 to investigate a 2cm neck mass identified in October 2018 as an incidental finding on a cervical spine magnetic resonance imaging (MRI) performed due to neck pain after a toboggan accident. The mass was associated with the inferior pole of the left thyroid lobe, but its precise anatomical origin was difficult to clarify. The patient did not describe dysphagia, dyspnea nor hoarseness of voice and the clinical examination didn’t reveal any cutaneous lesion. The medical history was notable for substituted hypothyroidism and renal colics between the age of 18 and 21; the latter had not been investigated further. The family history was unremarkable, in particular no history of NF1. The lesion had already been subjected to two ultrasound-guided fine-needle aspirations (US-FNA) performed by a head and neck specialist (both non-diagnostic) and a third one by an endocrinologist (non-diagnostic, with a mention of colloid).

### Clinical findings and timeline

**Figure 1:**
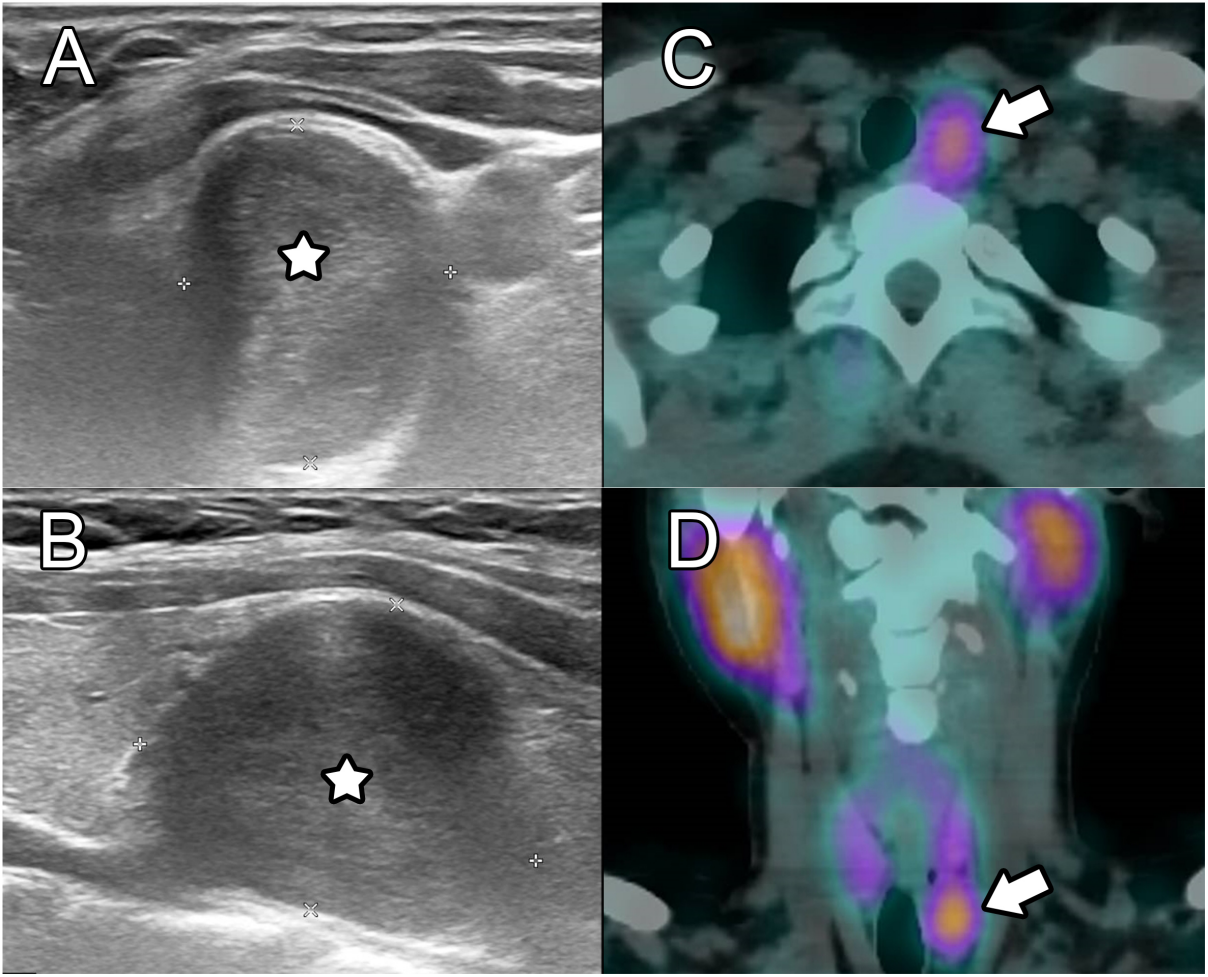
Initial ultrasound and SPECT/CT. Ultrasound on axial **(A)** and sagittal **(B)** views showed a well delineated hypoechoic round lesion adjacent to the inferior left thyroid lobe and left common carotid artery. On ^99m^Tc-sestamibi SPECT/CT, avid uptake was demonstrated on axial **(C)** and coronal **(D)** planes, which was initially thought to be consistent with a parathyroid adenoma.

Physical examination showed a palpable painless nodule below the left thyroid lobe that was mobile during swallowing. A new ultrasound was performed in January 2019, showing a 2.6 cm hypoechoic mass (4.5 ml in estimated volume) located posterior to the inferior pole of the left thyroid lobe, with scant vascularity (**Figures 1A, B**). Due to a clear visualization of a hyperechoic capsule between the mass and the adjacent thyroid parenchyma, the mass was suspected to be of extra-thyroidal origin, potentially parathyroid. On the contralateral side, a 0.8 cm mass was identified, compatible with a parathyroid gland or lymph node.

Although the patient did not present primary hyperparathyroidism biologically, a parathyroid origin could not be fully excluded because rare cases of nonfunctional parathyroid adenomas (4) and carcinomas (5) have been described, and such lesions can exhibit ^99m^Tc-sestamibi uptake (6). A SPECT/CT was thus performed in January 2019, showing ^99m^Tc-sestamibi avidity only in the larger nodule, which was negative for pertechnetate (**Figures 1C, D**). This pattern was suspicious for a parathyroid mass without biological function, or alternatively for a hyperplastic thyroid nodule, although lymph node metastasis from differentiated thyroid carcinoma or carcinoma of the head and neck, lung or breast can also show ^99m^Tc-sestamibi uptake. Other rare lesions exhibiting ^99m^Tc-sestamibi uptake include paragangliomas (7). However, these normally appear as pulsatile masses and show markedly internal vascularity on Doppler ultrasound (8) as well as a salt-and-pepper pattern on MRI (9).

The patient then underwent a new US-FNA, with measurement of multiple markers in the needle washout: parathyroid hormone (PTH), thyroglobulin (Tg) and calcitonin (Ct). Though cytology was again non-diagnostic, the levels of all three markers in the needle washout were below their respective detection thresholds (PTH <3 ng/l, Tg <0.15 ng/ml, Ct <0.5 ng/l), arguing against a parathyroidal or thyroidal origin of the mass. A core needle biopsy (CNB) was then performed, revealing spindle-shaped cells showing strong expression of S100 and SOX10 while lacking immunoreactivity for CD34, melan A, HMB45, cytokeratin, TTF1, and smooth muscle actin, consistent with the diagnosis of a schwannoma. Upon review of the MR images, and in view of its retrothyroidal localization, it was considered to arise from the left recurrent laryngeal nerve (RLN). The clinical evaluation was completed with a vocal cord endoscopy in March 2019 that showed normal functionality.

**Figure 2:**
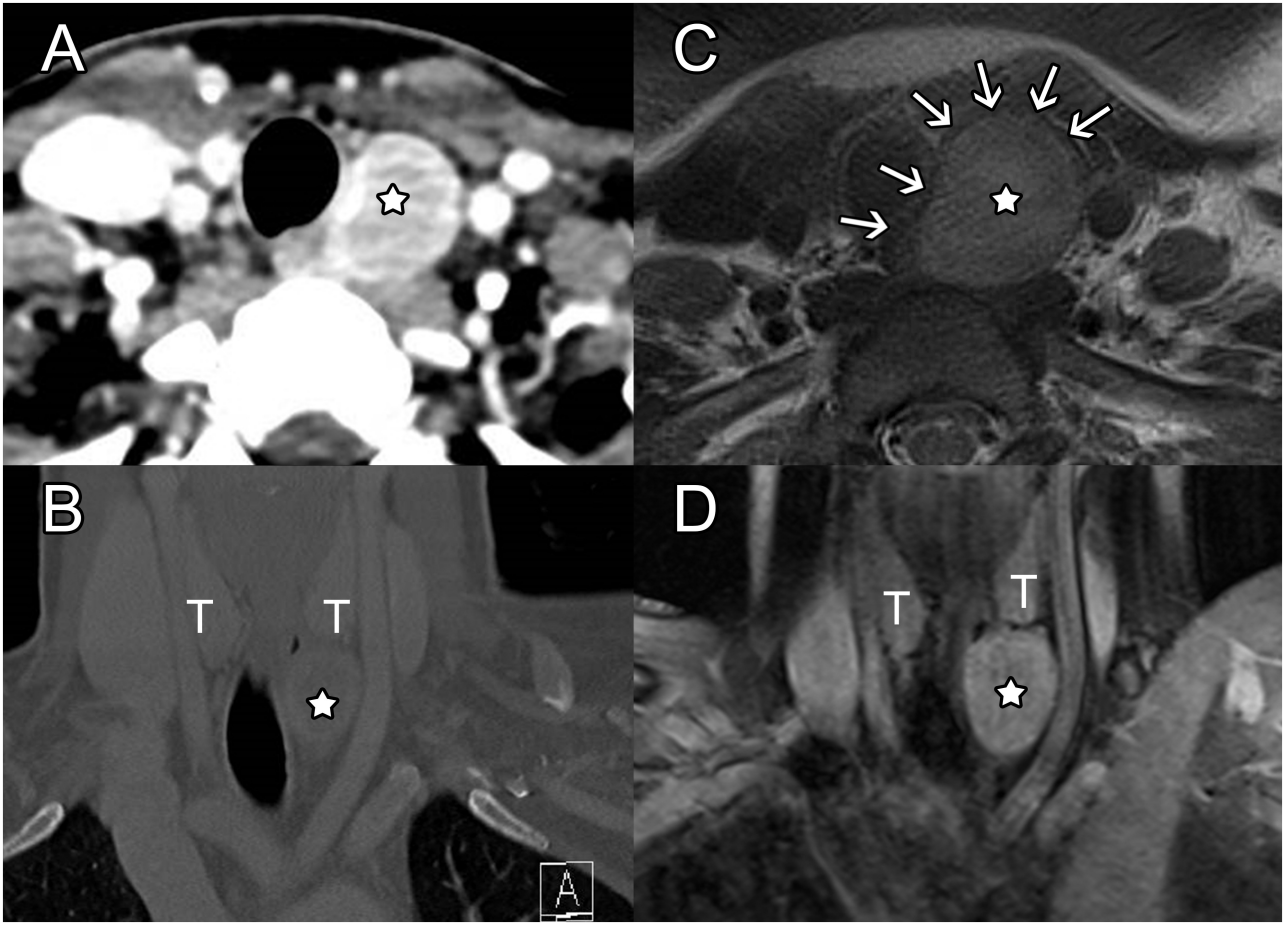
Follow-up CT and MRI. On axial **(A)** and coronal **(B)** contrast-CT views, the lesion again appeared well delineated with moderate enhancement and only a slight mass effect on the inferior aspect of the left thyroid lobe, on the left common carotid artery (CCA) and trachea. Perilesional fat was normal, and no dysplastic vessel was seen within the tumor. Axial T2-weighted MR images **(C)** highlighted that a thin hypointense rim (arrows) in continuity with the esophageal external layer wrapped the lesion. Homogeneous moderate enhancement was also seen on post-contrast coronal fat saturated T1-weighted images **(D)**. 7-month follow-up MRI demonstrated slow growth of the mass, which increased the lateral shift of the left CCA and the medial shift of the trachea without any sign of invasion.

Further revision of all images available from the past 3 years showed that the mass had clearly grown, with an estimated volume doubling time of approximately 1 year (352 days) (**Figure 2**). Therefore, surgical resection of the mass was strongly recommended, but the patient insisted on radiological surveillance instead. The next MRI was performed about 7 months later, showing a further increase in volume (**Figure 2**). The tumor exerted a mass effect on the trachea with a slight deviation to the right, without signs of infiltration or clinically relevant compression. The patient finally agreed to undergo surgical treatment.

### Therapeutic intervention

**Figure 3:**
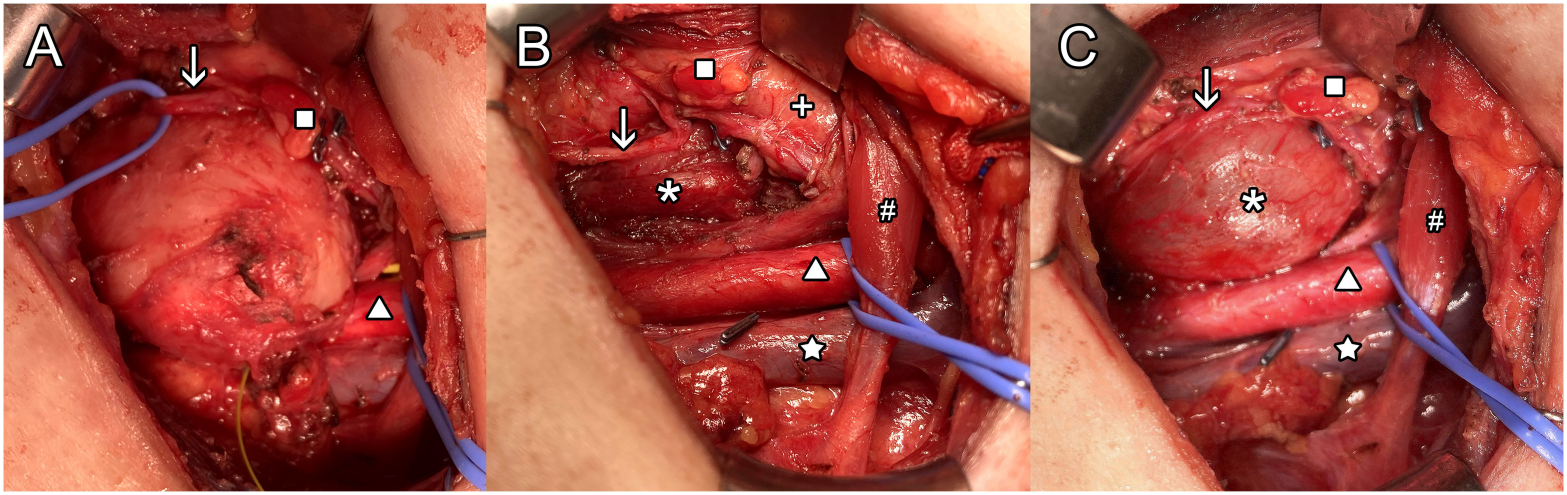
Surgical field by left cervicotomy approach. The greyish mass is well exposed in the center of the field **(A)** framed by the common carotid artery (CCA) inferiorly and the left recurrent laryngeal nerve (RLN) passing superficially. After enucleation **(B)** all structures seems intact except for the esophagus’s adventitia and muscularis propria layers. During esophageal insufflation, no perforation was visualized **(C)**. Labels: ↓ left RLN, Δ CCA, ☆internal jugular vein, □ inferior left parathyroid, * esophagus submucosal layer, # omo-hyoidien muscle, X thyroid left lobe.

**Figure 4:**
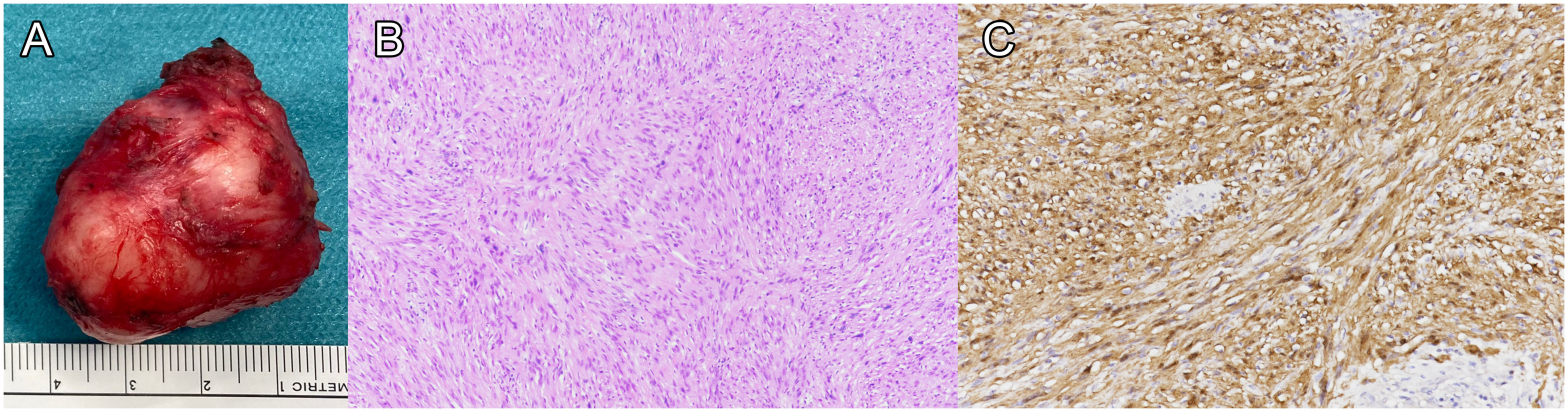
Histological analysis of the specimen. The specimen is shown after enucleation, without effraction of the mass **(A)**. Histological examination showed a proliferation of spindle-shaped cells with eosinophilic ill-defined cytoplasm and irregular, elongated nuclei without mitosis or necrosis **(B)**, hematoxylin-eosin stain). Tumor cells were strongly positive for S100 **(C)**.

Surgical resection was primary planned for a left RLN schwannoma, with possible sacrifice of the nerve and immediate non-selective reinnervation with a motor branch of the ipsilateral ansa cervicalis under continuous neuromonitoring of the left vagus-recurrent loop (10). After perfusion of a prophylactic dose of 2.2 g of co-amoxicillin, surgery started with a 5 cm left incision below the level of the cricoid cartilage. The internal jugular vein, internal carotid artery and vagus nerve were largely exposed and the automatic periodic stimulation (APS) clip was placed around the left vagus nerve. Continuous neuromonitoring showed a good signal with normal amplitude and latency. The mass was then exposed and the RLN was identified lying on it, adherent to its capsule. The RLN was carefully dissected from the capsule of the schwannoma without effraction (**Figure 3A**). Despite anatomic integrity of the RLN, APS monitoring showed increased latency and decreased amplitude without recovery, indicating an impairment of conduction within the dissected RLN (11). Dissection of the schwannoma was continued, with special care to avoid any capsular effraction. Surprisingly, the schwannoma was surrounded by stretched longitudinal and deeper transversal muscle fibers corresponding to the two muscular layers of the esophagus. Ultimately, the schwannoma was detached from the submucosal layer of the esophagus (**Figures 3B, C**, **Figure 4A**). Mucosal perforation was ruled-out by the absence of air leak while insufflating the esophagus through a naso-oesophageal tube with a pressure of 30 cm H_2_O (**Figure 3C**). The left inferior parathyroid gland was also preserved with its vascularization during the dissection (**Figure 3B**). The esophageal wall external longitudinal and internal circular muscular layers were closed with a running suture of 3-0 Vicryl and the neck was closed as usual with an aspirating drain. Extubation was uneventful. The patient resumed a liquid diet the evening of the surgery, and despite a clear hypomotility of the left vocal cord, she did not experience aspiration, but reported only mild dysphonia. Pain was efficiently controlled with paracetamol and ibuprofen.

In view of the intraoperative findings, retrospective review of the MRI confirmed that the schwannoma had developed within the wall of the esophagus, between the hyperintense submucosa and hypointense muscularis propria on T2-weighted images (**Figure 2C**).

Histological examination of the resected tumor showed an encapsulated nodule composed of compact bundles of spindle-shaped cells with eosinophilic ill-defined cytoplasm and irregular, elongated nuclei, some of which were enlarged and pleomorphic. Tumor cells grew forming compact areas (Antoni A pattern) with zonal palisading (Verocay bodies). Neither mitosis nor necrosis were present. Immunohistochemistry showed overlapping features with those observed in the pre-operatory biopsy, including diffuse and strong positivity for S100 and SOX10 and no expression of neurofilament, CD34, and smooth muscle actin. The Ki67 proliferative index was 5% (**Figures 4B, C**).

### Follow-up and outcomes

At the first follow-up evaluation 10 days after surgery, the patient reported improving mild cervical pain, occasional minor aspirations only with clear liquids, and persistent vocal fatigability without clear dysphonia. Clinical examination found a persistent hypomotility of the left vocal cord with good contralateral compensation, for which voice therapy treatment was started. At the last follow-up, four months after surgery, the patient reported complete resolution of all her postoperative symptoms, including voice fatigability and occasional aspirations. Indirect laryngoscopy showed complete resolution of the left vocal cord paresis with strictly symmetric and normal vocal cord movements.

## Discussion

Schwannomas are slow-growing encapsulated tumors, composed of Schwann cells and they are the most common peripheral nerve sheath tumors (PNSTs). They affect men and women equally and they can present at any age and at virtually any location in the body (12). In 25-45% of cases, they are located in the head and neck region (13). Schwannomas of the gastrointestinal (GI) tract are rare, and most of them are found in the stomach. Because of the even rarer nature of schwannomas originating from the esophagus, a correct preoperative diagnosis is often difficult to establish. Esophageal schwannoma develops more frequently in middle-aged women and is often located in the proximal esophagus. The main complaint is dysphagia (53.7%); less frequent symptoms include dyspnea (10.4%), cough (4.4%), weight loss (4.4%), chest pain (4.4%) and others. The tumor can also be asymptomatic, with a palpable mass being the only manifestation (14).

The use of imaging to diagnose schwannoma in the neck can be challenging. US is well-established as a convenient, accessible, safe and affordable tool for evaluating neck masses. Schwannomas usually appear on ultrasound as hypoechoic solid masses, but they can sometimes be cystic, have posterior acoustic enhancement, show a target appearance (hyperechoic center and hypoechoic periphery), or even show an internal vascular flow (15). The cystic appearance can be particularly challenging, as the differential diagnosis includes thyroid cysts and brachial cleft cysts (16). Among the various imaging options, MRI allows to establish the tissue of origin as well as provide additional information for the differential diagnosis. The so-called target sign (not present in our patient) has been described to be 100% specific for schwannomas, with a sensitivity of 59%; it is defined as a biphasic pattern with higher intensity peripherally and lower intensity centrally on T2-weighted images, and higher intensity centrally by focal enhancement and lower intensity peripherally on gadolinium-enhanced T1-weighted images (17). MRI features that raise suspicion of malignancy are ill-defined margins, invasion of fat planes, and peritumoral edema (18). When malignancy is suspected, imaging by 18F-fluorodeoxyglucose positron emission tomography/computed tomography (^18^F-FDG-PET/CT) can also be helpful to complement MRI in discriminating benign from malignant PNSTs, ^18^F-FDG-PET/CT being more sensitive than MRI (90.0-100.0% *vs*. 62.5-81.3%) but less specific (52.2-82.6% *vs*. 94.1-100.0%) (19).

We searched the literature for previous cases of ^99m^Tc-sestamibi uptake by a cervical schwanoma. The only potentially relevant article reported the case of a 53-year-old patient with primary hyperparathyroidism, who, after multiple negative localization tests (^99m^Tc-sestamibi scan, cervical ultrasound and cervical MRI), had removal of both left parathyroid glands (20). Three years later, because of persistent hyperparathyroidism, a new ^99m^Tc-sestamibi scan was performed showing uptake in the right cervical region, followed by a cervical CT scan that revealed a 1.1 cm nodule between the jugular vein and the right carotid. At this location, a 1.5 cm tumor was identified during surgery and histology showed a schwannoma of the vagus nerve. Although the authors describedthe schwannoma as behaving like a mimicker of a parathyroid adenoma, we could not find clear evidence of ^99m^Tc-sestamibi uptake by the schwanoma (as opposed to by a parathyroid adenoma responsible for the patient’s persistent hyperparathyroidism), because no SPECT/CT scan was done and no ^99m^Tc-sestamibi scan images were shown in the article (20).

In the neck region, ^99m^Tc-sestamibi activity is commonly associated with parathyroid disease; in at least one-third of patients with hyperparathyroidism, it is also associated with thyroid abnormalities (nodules or autoimmune disease) (21, 22). Uptake and prolonged retention has also been described in various primary and metastatic tumors, as well as in inflammatory, infectious (abscess) and granulomatous (tuberculosis, sarcoidosis) diseases, which can lead to false-positive results when searching for parathyroid lesions (1). Outside the neck, uptake of ^99m^Tc-sestamibi in schwannomas has already been described as a rare finding in the head (2), and to some extent it has been shown to be an effective radionuclide in the localization of primary and metastatic brain tumors and acoustic schwannomas (^99m^Tc-sestamibi brain SPECT of cerebellopontine angle tumors). It has also been reported as an incidental finding, identifying a gastric schwannoma in a patient investigated for primary hyperparathyroidism (23).

FNA was not helpful in our case to establish the diagnosis, even though measurement of thyroid and parathyroid markers in the needle washout was helpful to exclude thyroidal and parathyroid origin of the mass. The challenge of non-diagnostic FNA has been reported by many others for extracranial head and neck schwannomas, with the main difficulty being to collect sufficient material (24). For example, one study reported that only 24 out of 52 FNAs (46%) were able to diagnose schwannomas, as compared to 28 out of 29 CNBs (97%) (25). In our case, the esophageal submucosal tumor was accessible for a core biopsy, but in most cases differential diagnosis of a gastrointestinal submucosal tumor (GIST, leiomyoma, neurofibroma, schwannoma, etc.) is rarely achieved preoperatively; in such cases, surgical resection is usually recommended due to symptoms, tumor size or complications directly linked to tumor growth (14). Complete surgical resection is often also needed for a definitive diagnosis and is the treatment of choice for non-vestibular head and neck schwannomas (26). The differential diagnosis of benign PNSTs comprises perineuromas, neurofibromas, hybrid nerve sheath tumors and others. Histology has a central role in differentiating among them, with typical schwannomas showing either a dense or a loose architectural pattern (called Antoni A or B, respectively), with cells presenting spindle-shaped nuclei. Immunohistochemical staining for S-100 protein is often strong and uniform, which helps in confirming the diagnosis of schwannoma (27). Albeit challenging in most cases, a pre-operative diagnosis of schwannoma grants the possibility to complete the imaging evaluation to pinpoint the tissue/nerve of origin, to discuss with the patient possible complications of surgery when resection is indicated, and to plan the procedure with appropriate monitoring to reduce morbidity (25, 28). In this specific case, we asked ourselves if a different surgical approach would have been chosen if we knew that the schwannoma derived from the submucosal layer of the esophagus. Indeed, endoscopic resection of submucosal esophageal schwannoma is possible and less invasive (29). However, this approach is limited to small tumors protruding within the esophageal lumen. In our case, the bulk of the tumor was outside the lumen (**Figure 3**) and the close proximity of the lateral side of the tumor to the area of the left RLN would have been considered a contra-indication to endoscopic resection.

In conclusion, this case illustrates that schwannoma should be considered in the differential diagnosis of a mass showing focal ^99m^Tc-sestamibi uptake in the neck region in the absence of biochemical evidence of primary hyperparathyroidism, and that CNB should be performed to guide further therapeutic management whenever US-FNA and cross-sectional imaging are unable to provide a diagnosis.

## Patient perspective

In October 2018, I discovered by chance while doing a MRI because of a toboggan accident I had in February 2017, that I had a small mass next to my thyroid. That was very hard for me because I already had an issue with my shoulder to resolve. During around 6 months, I had five biopsies because I did not want to be operated without knowing what it really was. After discovering that I had a schwannoma, and knowing that it was a benign tumor, I always had hope that I would not need surgery. I was very afraid of the procedure, of a permanent visible scar, and of the possibility of a permanently altered voice. I had an MRI every 6 months to check the tumor’s size. It grew a lot during 2 years. I knew in my head that I had to make the decision to have surgery, but the fear was an obstacle. In May 2020, I made the decision to have surgery because the tumor had grown a lot. I knew that I had no choice anymore. It was a very difficult decision for me. When I made the decision, I felt that I needed professional help to prepare myself for the operation. I had consultations with a psychiatrist that helped me a lot. The day before the operation, I was feeling in peace and very serene because I just wanted to remove the tumor and be able to continue living my life. In the morning, when I woke up, I was sure that I was ready and very confident. Before the operation, already in the operating room, I felt reassured. When I woke up from the anesthesia and was able to speak, it was a great joy for me because my voice was not altered. After the operation, everything went well, without much pain, just a small bother due to the feeding tube. Today, I regret not having made the decision earlier because I suffered a lot for 2 years. I am very glad I had this surgery and I thank the surgeon for always having reassured me.

## Data availability statement

The original contributions presented in the study are included in the article/supplementary material. Further inquiries can be directed to the corresponding authors.

## Ethics statement

The studies were conducted in accordance with the local legislation and institutional requirements. The participants provided their written informed consent to participate in this study. Written informed consent was obtained from the individual(s) for the publication of any potentially identifiable images or data included in this article.

## Author contributions

RF: Writing – original draft. EG: Writing – original draft. SL: Writing – review & editing. VD: Writing – review & editing. GS: Writing – review & editing. FG: Writing – review & editing.
